# Acceptance of Human Papillomavirus Vaccination and Associated Factors among Girls in Arba Minch Town, Southern Ethiopia, 2020

**DOI:** 10.1155/2022/7303801

**Published:** 2022-12-07

**Authors:** Eshetu Y. Ukumo, Feleke G. Weldehawariat, Samuel A. Dessalegn, Desta M. Minamo, Haymanot N. Weldehawaryat

**Affiliations:** ^1^Department of Midwifery, College of Medicine and Health Sciences, Arba Minch University, Arba Minch, P.O. Box 21, Ethiopia; ^2^School of Public Health, College of Medicine and Health Sciences, Arba-Minch University, Arba-Minch, P.O. Box 21, Ethiopia

## Abstract

**Background:**

Cervical cancer is Ethiopia's second biggest cause of cancer-related death among women. The introduction of human papilloma virus (HPV) vaccination is expected to have a significant impact on the burden of cervical cancer. In Ethiopia, particularly in our study area, little is known regarding girls' acceptance of HPV vaccination. Therefore, this study has assessed the acceptance of HPV vaccination and associated factors among girls in Arba Minch town, southern Ethiopia.

**Methods:**

A school-based cross-sectional study was conducted on January 1, 2020. Based on convenience, Arba Minch town was purposefully selected. Stratification was done to stratify private and public schools, then simple random sampling to select sample schools from each, and finally, a proportional allocation of sample size to each school. The determinants and independent variables that influence the acceptance of the human papillomavirus vaccination were determined using a multivariable logistic regression model.

**Results:**

This study's overall acceptance rate for study participants was 50.4% (95% CI) (45.9–55.2). Girls' age (AOR = 2.93, 95% CI (1.57_5.47), *P* value 0.001), mothers' educational level (secondary and more than secondary, AOR = 2.40, 95% CI (1.01_5.73), *P* value 0.048, and 3.64, 95% CI (1.61_8.25), *P* value 0.002, respectively), positive attitude (AOR = 5.22, 95% CI (2.96_9.19), *P*value ≤ 0.001), good knowledge (AOR = 2.49, 95% CI (1.19_5.24), *P* value 0.001), and receiving childhood immunization (AOR = 14.85, 95% CI (8.58_25.72), *P*value ≤ 0.001) were factors associated with girls' acceptance of the human papillomavirus vaccination. *Conclusions and Recommendation*. Only half of the study participants accepted HPV vaccination. Therefore, Arba Minch town health institutions should better boost the acceptance of HPV vaccination by improving the knowledge and attitudes of girls. Factors associated with girls' acceptance of HPV vaccination were age, mothers' educational status, positive attitude, knowledge of HPV vaccination, and receiving childhood immunization.

## 1. Background

After breast, colorectal, and lung cancers, cervical cancer is the fourth most common malignancy in women and one of the greatest threats to a woman's life [1]. Cervical cancer is responsible for 311,365 deaths and 569,847 new cases worldwide each year. The most common human papillomavirus types 16 and 18, which account for over 70% of all cervical malignancies, are those that cause invasive cervical cancer. 85 percent of cervical cancer diagnoses worldwide occur in impoverished countries [2]. The greatest cancer mortality rates among women worldwide are found in sub-Saharan Africa, where cervical cancer incidence rates are also among the highest in the world. The high prevalence of cervical cancer is a result of most nations' incapacity to start or maintain services for cervical cancer prevention. Additionally, it seems that women with normal cytology have a greater prevalence of HPV, at an average of 24%, than women do in more industrialized regions of the world. However, there are considerable regional differences in sub-Saharan Africa, with Eastern and Western Africa having the highest rates of HPV infection and cervical cancer [3]. In Ethiopia, it is predicted that 4,732 women die from the disease each year and 7,095 women are diagnosed with cervical cancer [4].

Cervical cancer is more common in lower resource areas; hence, the introduction of HPV vaccination offers a way to minimize the disease's burden [5–7]. Prophylactic HPV vaccination, when administered prior to sexual activity, provides nearly 100 percent protection against persistent infection with vaccine-targeted high-risk HPV strains (e.g., HPV-16 and 18) and associated precancers [8]. Human papillomavirus vaccination has the potential to improve, strengthen, and integrate health services for adolescent females at the national, regional, and local levels [2]. However, many countries' acceptance of HPV vaccination is low [9–14]. Vaccine apprehension is multifaceted and situational. Some of the reasons for vaccine hesitancy are risk-benefit analysis (scientific evidence, i.e., vaccine safety concerns and fear of side effects), a lack of knowledge and awareness of vaccination and its importance, and a lack of knowledge of parents on the benefits of immunization, religion, culture, gender, and socioeconomic issues regarding vaccines [15]. As a result, every government must take consistent steps to identify the scope and nature of the reluctance. Countries should also devise a strategy to boost acceptability, trust, overcome hesitation, and plan for crisis reaction.

Factors that affect the acceptance of HPV vaccination among girls include age, parent education, place of getting the HPV vaccination, knowledge of HPV vaccination, discussion with health care providers, belief that HPV vaccination can prevent HPV infection, type of school, school setting with a reproductive health club, effectiveness and negative attitude toward HPV vaccination, completion of childhood vaccination, the advancement of reproductive health services and peer role, peer encouragement, and fear of needles [12, 16–29].

The introduction of a primary preventive vaccination is anticipated to have a major influence on the burden of cervical cancer, especially in areas where screening is lacking, limited, or of poor quality [30]. With the help of the global alliance for vaccines and immunization, in December 2015, Ethiopia launched a pilot HPV vaccination project in Gomma Woreda (District) of Jimma Zone in Oromia Region and Ahferom Woreda (District) of Tigray Region in December 2015, targeting adolescent girls in the 9–13-year age group. In December 2018, Ethiopia launched a nationwide human papillomavirus (HPV) vaccine through a school-based approach to reach all eligible girls in both private and public schools. For out-of-school girls, the vaccine is given at any health facility in all 11 regions and the two city administrations of the country [31]. On the other hand, vaccination for vulnerable girls and women in Ethiopia faces a number of challenges, including vaccine shortages, insufficient delivery infrastructure, erroneous beliefs on the origin and treatment of cervical cancer, and a lack of community engagement to raise awareness about cervical cancer and early screening tools [7, 32–36]. Therefore, country-specific data is very important to follow the achievements and identify obstacles to newly commenced programs.

For us to be sure about what is unknown in Ethiopia and in the study area about the HPV vaccination of girls as well as to introduce the factors that affect the acceptance of the girls' HPV vaccination in Ethiopia and in the study area, there is no study preceding this study in our study area as well as other parts of the country. Therefore, this study assessed the acceptance of the HPV vaccination and associated factors among girl students in Arba Minch town, Southern Ethiopia. The result of this study is a crucial source of information for health program planners, especially for those who work to decrease cervical cancer prevalence and further investigators.

## 2. Materials and Methods

### 2.1. Study Setting and Design

The study was carried out in Arba Minch town, which is 505 kilometers from Addis Abeba and 280 kilometers from Hawassa. This study used a cross-sectional study design and was conducted on January 1, 2020. The data collection was conducted within one day by assigning different data collectors and data collection supervisors to each of the selected schools in order to avoid data contamination. But, training for data collectors and supervisors was given before data collection day.

For the sake of convenience in financial- and time-related terms, this study was conducted in Arba Minch town. We studied only acceptance of HPV vaccination, leaving others, such as knowledge and attitude of girls toward HPV vaccination, as independent variables. This was because we could not find enough references regarding the knowledge and attitude of girls toward HPV vaccination. We focused our study only on HPV vaccination because it was a newly commenced program, and cervical cancer is our country's most prevalent health problem. We focused our study on school girls because almost all girls eligible for HPV vaccination can be found in schools, and we also wanted to conduct an institution-based cross-sectional study. We did not conduct our study in all adolescent groups because only age groups 9 up to 14 were eligible for HPV vaccination.

### 2.2. Inclusion Criteria

Those girls who were between 13 and 14 years old were selected. This age group was selected because the human papillomavirus vaccination was temporarily applicable only to this age group during our data collection period in this study area due to the national vaccine shortage. So, we wanted to study acceptance of HPV vaccination in the age group in which vaccination is applicable because it seemed logically impossible to us to study acceptance in the age group where vaccination is temporarily not applicable.

### 2.3. Exclusion Criteria

Those girls who did not agree to participate in the study.

Those girls whose families/guardians/relatives are deprived of their participation through assent form.

### 2.4. Sample Size Determination

The sample size was determined by using a single population proportion formula with the assumption of a 95% confidence level and a 5% margin of error, by taking the “proportion of acceptance of HPV vaccination =0.669” from similar studies, especially by study design, study participants, and study place [20], using a 1.5 design effect, and then adding a 10% nonresponse rate. The total number of participants was 561.

### 2.5. Sampling Technique

**Figure 1:**
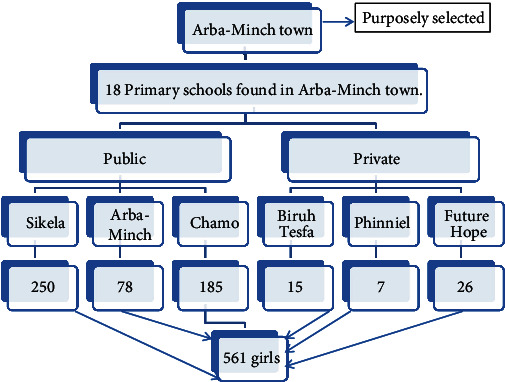
Sampling procedures to select study units from primary schools in Arba Minch town, Southern Ethiopia, January, 2020.

First of all, we selected Arba Minch town purposely. In Arba Minch town, there were 18 primary schools at which eligible students were present. They were then stratified into public and private. Then, we selected six schools, three schools from each, using a simple random sampling technique. Then, the calculated sample size was allocated to each school proportionally. We prepared a sampling frame for each selected school. Then, study units of a given school were obtained by a simple random sampling technique. Finally, selected study units for a given school were asked to be in one place and fill out the questionnaire through the guidance of data collection facilitators and supervisors (Figure 1). Data collectors were selected from among the teachers of their respective schools. Then, using a straightforward random sampling procedure, study participants from a certain school were collected. Finally, under the supervision of facilitators and supervisors for data collection, selected participants for a certain school were requested to assemble in one location and complete the questionnaire. Data gatherers were picked from among the teachers at their respective schools. Supervisors were selected from instructors at the Arba Minch University.

### 2.6. Data Gathering Device

A self-administered, structured questionnaire that had been pretested was employed. It was also translated into Amharic. The device we used to assess the acceptance of HPV vaccination was prepared by a thorough review of related articles. Adolescent girls in Arba Minch town are asked to answer questions about their sociodemographic characteristics, attitudes, knowledge, and acceptance of the HPV vaccine. To avoid leaving out crucial information, the tool was written in English and then translated into Amharic. The text was subsequently retranslated into English for study.

### 2.7. Data Quality Assurance

The training included the purpose of the study, the time frame for data collection, the timely gathering and reorganization of the collected data from individual schools, and the submission deadline. The questionnaire was pretested in another elementary school that was not used in this study with 5% of the sample size. Final data was gathered from devices having a Cronbach's alpha value of 0.7 and higher. The quality of the data was regularly checked while collecting the data and during data input, in addition to the proper recruitment and training of the data collectors. Throughout the data gathering process, every questionnaire was checked for accuracy and consistency.

### 2.8. Data Processing and Analysis

EpiData version 4.6 was used to enter the data, which was then exported for analysis in SPSS version 23. The results of a descriptive study were used to calculate a central tendency index.

For the purpose of calculating knowledge scores, each accurate response received one point, while inaccurate or “do not know” responses received zero. The respondents' degree of knowledge was then categorized based on their score, and the results of the overall seven knowledge questions after the total knowledge score were translated into percentages. Participants in the study who achieved a 50% score (i.e., correctly answered three or fewer questions) were classified as having low knowledge, and those who achieved a 50% score or higher were classified as having good knowledge [16]. A 5-point Likert scale ranging from “strongly disagree” to “strongly agree” was used in scoring attitudes toward the HPV vaccination. One point was given to “strongly disagree” responses, two points for “disagree” responses, three points for “no opinion” responses, four points for “agree” responses, and five points for “strongly agree”. For negative questions, the scoring system was reversed. The total attitude score was then converted into percentages and classified as follows: a score of 50.0 to 100.0% represented a “positive attitude, “ while less than 50% was judged as a “negative “[37]. Acceptance was also assessed by using a 5-point Likert scale ranging from “strongly disagree” to “strongly agree”. Based on the total score, a cut-off point of 50 points or higher was used; any score above 50 was considered accepted the vaccination, and any score below that was considered not accepted the vaccination [38].

For the purpose of examining the relationship between the dependent and independent variables, multivariable logistic regression was used. To reduce potential confounders, independent variables having a *P* value of less than 0.25 were added to the multivariable model. Using a variance inflation factor, the multicollinearity test was performed to determine the correlation between the independent variables. Independent variables with a *P* value of 0.05 or less in the multivariable logistic regression model were considered as statically significant factors for acceptance of the HPV vaccination. The results were presented as odds ratios (OR) with 95% confidence intervals. The model fitness was checked by using the software application of the Hosmer and Lemeshow tests (>0.05). The results were presented using text and tables.

## 3. Results

### 3.1. Sociodemographic Characteristics of Study Participants

Only 516 of the 561 people who received samples could answer, yielding a 92% response rate. Forty-five surveys were excluded from the analysis due to missing data. More than half of the participants were 14 years old (61%). The majority of study participants (60.5%) were Protestant Christians. 36.63% of the study participants were in grade 8. About 20.3% of respondents had maternal education only (Table 1).

### 3.2. Knowledge on HPV Vaccination and Related Responses

Three hundred ninety-six (75.2%), 95% CI: 71.5-79, of the study participants had an excellent understanding of HPV vaccination, and it was discovered. 69.8% of survey participants were aware of the recommended age for HPV vaccination. For 69.8% of study participants, their main source of information was social media. Sixty-one percent of respondents (60.1%) knew that the HPV vaccination is given for the prevention of cervical cancer (Table 2).

### 3.3. Attitude of Girl Students towards HPV Vaccination

The overall attitude of the study participants towards the HPV vaccination was 51%, 95% CI, and 46.9_55.4. 68.4% of the study participants believe that “HPV vaccination should be delivered before starting sexual activity.” Less than three-quarters, 61.7%, of the study participants believe that “HPV vaccination can prevent cervical cancer.” 16.5% “agree” that the “HPV vaccination is only for people who are sexually active” (Table 3).

### 3.4. Acceptance of HPV Vaccination

**Figure 2:**
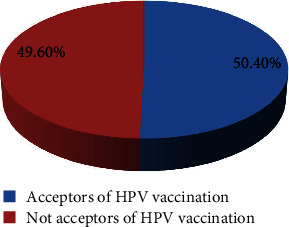
Level of acceptance of HPV vaccination among girl students in Arba Minch town, Southern Ethiopia, 2020.

Students preferred to be vaccinated against HPV at school (67.44%), followed by health institutions (20.54%) (Table 3). Two hundred sixty (50.4%) CI 95% (45.9-55.2) of the study participants accepted the HPV vaccination (Figure 2). 64.7% of students agreed to receive the HPV vaccination for them if it was available, and 57.9% agreed to receive the HPV vaccination for their friends (Table 4).

### 3.5. The Link between Religion, Ethnic Group, and the Acceptability of the HPV Vaccination

The Muslim religion's followers had a much higher proportion of acceptors (11, 64.7%). Wolayta ethnic grouping 21 (63.6%) had the highest proportion of acceptors in the etheric group. The HPV vaccination was accepted by 87 (58.8%) of the Orthodox religion's followers. 140 (44.9%) of protestant religious followers accepted HPV vaccination (Table 5). Individual disparities in information access and attitudes concerning HPV vaccination may be to blame for this.

### 3.6. Factors Determining the Acceptance of HPV Vaccination

Multivariable logistic regression analysis was used to identify the statistically significant factors associated with the acceptance of the HPV vaccination. Those significant factors were the age of girls, mothers' educational status, having childhood immunization, attitude, and knowledge level about the HPV vaccination.

Those who were 14 years old were close to threefold more likely to accept the HPV vaccination as compared to those who were 13 years old (AOR = 2.93, 95% CI (1.57_5.47), and *P*value = 0.001).

Those girls with mothers' educational status of secondary and more than secondary were more likely to accept HPV vaccination than those in primaries and below (AOR = 2.40, 95% CI (1.01_5.73), *P*value = 0.048, 3.64, 95% CI (1.61_8.25), and *P*value = 0.002, respectively). There were nearly fifteen times as many people who got their childhood vaccinations as those who had not accepted the HPV vaccine (AOR = 14.85, 95% CI (8.58_25.72), and *P* value 0.001). Positive attitudes were nearly five times more likely than negative attitudes to accept HPV vaccination (AOR = 5.22, 95% confidence interval (2.96_9.19), and *P*value ≤ 0.001. When compared to individuals with inadequate awareness, those with high information were nearly twice as likely to accept the HPV vaccination (AOR = 2.49, 95% CI (1.19_5.24), and *P* value is 0.016 (Table 6).

## 4. Discussion

In this study, the overall acceptance of HPV vaccination was 50.4%, which was lower than the results from Jinan, China (66.9% [20]), India (74.4% [39]), and Los Angeles (62% [23]) but higher than the results from Hong Kong, China (36.5% [40]), the Netherlands (39% [41]), and Turkey 11.2% [42]. From African countries, it is less than Mali's 100% [9], Mozambique's 91% [43], and more than Senegal's 28% [13]. This might be due to the difference in the definition of the acceptance of HPV vaccination, study subjects involved, socioeconomic status, health information access, and tools used to measure the acceptance of HPV vaccination. The acceptance of the HPV vaccination in this study was only half, which might be due to poor health information access, less encouragement of girls to participate in school seminars and health-related school clubs, and sociocultural influence on female gender.

The age of study participants was significantly associated with their acceptance of the HPV vaccination. That is, those who were 14 years old were threefold more likely to accept the HPV vaccination as compared to those who were 13 years old. This is similar to a study conducted in Melaka, Malaysia [44] but different from a study conducted in Uganda, where there was no statistically significant association between age and acceptance of HPV vaccination [22]. It might be due to methodological and sociodemographic differences. The association might be due to increasing age, which may be associated with increased education level, which in turn may be associated with increased access to health information access and involvement in different school seminars. Not only this, but also in the different communities in Ethiopia, increasing age gives the right to access and take part in some activities. Activities can be of any type, such as giving the chance of access to health information and involvement in different seminars.

Positive attitudes were nearly five times more predictive of HPV vaccination acceptance than negative attitudes. This study is comparable to one that was done in Jinan of China [20] and Los Angeles [23]. The positive relationship between attitude level and acceptance of HPV vaccination may be due to the level of awareness of respondents about the risk of cervical cancer, their perception of the potential risk of exposure to it, and good health-seeking behavior.

The likelihood of accepting the HPV vaccination is about two times higher for people with adequate information than for those with poor knowledge. This is similar to a study conducted in Melaka, Malaysia [44] but different from a study conducted in Ibanda District in Uganda, in which there was no positive association between knowledge of the HPV vaccination and its acceptance [24]. This could be due to methodological and sociodemographic differences. The higher degree of awareness of the study participants regarding the significance of HPV vaccination, the danger of cervical cancer, and their favorable attitudes toward HPV vaccination may account for the association between good knowledge and acceptance of the HPV vaccination.

Taking childhood immunizations was also associated with acceptance of the HPV vaccination. Those who had received their childhood immunizations were approximately fifteen times more likely than those who had not received their childhood immunizations to accept the HPV vaccination. This research is comparable to one done in Australia [25]. The connection could result from the fact that those who took childhood vaccinations may have a positive attitude toward the vaccinations and may have good access to information sources about the vaccinations.

With their mothers' level of education, daughters are more likely to accept the HPV vaccine. In other words, those whose moms had secondary, nearly twice as high, and more than secondary education were, accordingly, about four times more likely to accept HPV vaccination than those whose mothers had elementary and lower education. This research is similar to one that was conducted in Melaka, Malaysia [44]; nonetheless, the research done in Senegal yielded the opposite result. Parental education status has a negative association with daughters' acceptance of HPV vaccination [13]. This may be due to attitude and cultural differences. The maternal education status association with their daughters' acceptance of the HPV vaccination in this study might be due to educated mothers' being an information source by openly discussing their daughters' health issues, including the HPV vaccination, the severity of cervical cancer, and the importance of immunization. Additionally, minorities are the main target group for HPV vaccination; as a result, children under the age of 18 are unable to make this option for them and are instead primarily influenced by their parents. The parents' level of education may influence whether they decide to allow their daughters to receive vaccinations, which may impact the acceptance rate of their daughters.

### 4.1. The Strength of the Study

The study's quality was undeniable due to the multidisciplinary approach it used to complete it. Additionally, a pretest was completed to assess the tool's applicability, reliability, and validity. It was able to poll a random sample of female students to boost the generalizability of the findings to other students. This study used a self-administered questionnaire, which improves internal validity by increasing the likelihood that respondents will answer honestly. Future academics, health officials, and program implementers can use the findings from this study as a starting point to further their understanding of teens' acceptance of the HPV vaccine following the implementation of a national HPV vaccination campaign.

### 4.2. Constraints of the Research

The constraints was the lack of a strong qualitative research to get a direct and deep understanding of girls' concerns about the HPV vaccination; some of the girls' important concerns might not have been included in this study. This study did not include the daughters' parents, who are the primary source of information for their young daughters and decision-makers for their daughters who are under 18 years old according to Ethiopian law. Therefore, the parents' access to sources of information, awareness, and attitude may deter their decision to accept vaccination for their daughters, which can affect the acceptance rate of their daughters. As a result, the next researcher should consider them to dig out their attitude, knowledge, and acceptance toward HPV vaccinations. Acceptance of HPV vaccination in this study was assessed hypothetically, which may not necessarily translate into actual acceptance during real interventions when the HPV vaccine is at hand. So, the coming investigator/s should study actual acceptance on the day of vaccination while HPV vaccination is at hand. The age cohort of this study was limited to 13 to 14 because only this age group was taking HPV vaccination in the study area. We were limited to studying acceptance at the age group at which vaccination was applicable. As a result, generalization to other age groups may be difficult, so future studies should consider other age groups when the national vaccine shortage is overcome.

## 5. Conclusion

In developing countries, including Ethiopia, where cervical cancer is the leading cause of death due to poor health-seeking behavior and limited supplies and access, HPV vaccination is the most important measure to decrease the economic, social, and health-related burdens of cervical cancer and improve the health and well-being of women. Despite this fact, only half of the study participants accepted HPV vaccination. Unless immediate actions are taken to improve the girls' acceptance of HPV vaccination, it may undermine the utilization of it. Factors associated with girls' acceptance of the HPV vaccination were age, mother's educational status, positive attitude toward HPV vaccination, knowledge of HPV vaccination, and receiving childhood immunization.

We recommend that Arba Minch town health care institutions and workers increase the acceptance of HPV vaccination by increasing knowledge and positive attitudes about the vaccination with the mobilization of health extension workers, health development armies, teachers, and school minimedia workers. It is also better if schools insure girls' participation in health-related seminars and clubs, as this in turn may improve their health-related information, including HPV vaccination.

Health information dissemination campaigns should include parents as their attitude and knowledge toward HPV vaccination may hinder their decision to accept vaccination for their daughters, which can affect the acceptance rate of their daughters. HPV vaccination targets are minor groups, i.e., those who are under 18 years old and unable to decide for themselves. This study provides crucial information for health program planners and implementers, especially for those who work to decrease cervical cancer prevalence and basic information for further investigators.

## Figures and Tables

**Table 1:** Sociodemographic characteristics of girl students in Arba Minch town, Southern Ethiopia, 2020.

Variables	Categories	Frequency (*n*)	Percent (%)
Age in years	13	201	39.00
	14	315	61.00

Religion	Protestant	312	60.50
	Orthodox	148	28.70
	Catholic	20	3.90
	Muslim	17	3.30
	Others	19	3.70

Ethnicity	Gamo	368	71.20
	Gofa	46	8.90
	Amhara	33	6.40
	Oromo	23	4.40
	Wolayta	33	6.40
	Others	13	2.50

Grade level	4 and5	58	11.20
	6	124	24.03
	7	145	28.10
	8	189	36.63

School type	Public	470	91.10
	Private	46	8.90

Mother's educational status	No education	112	21.70
	Primary education	115	22.30
	Secondary education	105	20.30
	More than secondary	184	35.70

Father's educational status	No education	72	14.00
	Primary education	89	17.20
	Secondary education	107	20.70
	More than secondary	248	48.10

**Table 2:** Knowledge of girl students about HPV vaccination and related responses in Arba Minch town, Southern Ethiopia, 2020.

Variable	Categories	Frequency (*n*)	Percent (%)
Do you know HPV vaccination	Yes	408	79.10
	No	108	20.90

What is HPV vaccination	Given for prevention of cervical cancer	310	60.10
	Given for prevention of malaria	50	9.70
	Given for prevention of HIV ADDIS	16	3.10
	I do not know	140	27.10

Cervical cancer screening is advisable after HPV vaccination	Yes	383	74.20
	No	133	25.80

HPV vaccination should be taken before starting sex	Yes	292	56.60
	No	224	43.40

Number of HPV vaccination	Twice	404	78.30
	Once	112	21.70

Age at which someone undergo HPV vaccination	9-14 years	360	69.80
	18-21 years	30	5.80
	All ages	27	5.20
	I do not know	99	19.20

An interval that someone take second HPV vaccination	6 month	314	60.90
	6 year	62	12.00
	1 month	26	5.00
	3 month	114	22.10

Childhood vaccination	Yes	266	51.60
	No	250	48.40

Place of preference to have HPV vaccination	School	348	67.44
	Health institution	106	20.54
	At home	62	12.02

Presence of any reproductive health-related clubs	Yes	159	30.80
	No	357	69.20

Sources of information	Media	357	69.20
	Health care worker	106	20.50
	Others(family and friends)	53	10.30

Knowledge level	Poor knowledge	128	24.80
	Good knowledge	388	75.20

**Table 3:** Attitude of girl students towards HPV vaccination in Arba Minch town, Southern Ethiopia, 2020.

Attitude-related questions of HPV and its vaccination	Strongly disagree	Disagree	Neutral	Agree	Strongly agree
Information from media encouraged me to be vaccinated against HPV	77 (14.90%)	81 (15.7%)	89 (17.2%)	86 (16.6%)	183 (35.4%)
Information from health workers encouraged me to be vaccinated against HPV	43 (8.3%)	96 (18.6%)	82 (15.9%)	70 (13.6%)	225 (43.6%)
My friends encourage me to take HPV vaccination.	50 (9.7%)	83 (16.1%)	77 (14.9%)	76 (14.7%)	230 (44.6%)
My family encourage me to be vaccinated against HPV	46 (8.9%)	74 (14.3%)	88 (17.1%)	57 (11%)	251 (48.5%)
I think HPV vaccination can prevent cervical cancer	37 (7.2%)	48 (9.3%)	113 (21.9%)	72 (14.0%)	246 (47.7%)
Parental culture hinders me from taking the vaccination	40 (7.8%)	33 (6.4%)	129 (25.0%)	108 (20.9%)	206 (39.9%)
In my religion, taking HPV vaccination to girls is forbidden	41 (7.9%)	30 (5.6%)	137 (26.6%)	108 (20.9%)	200 (38.8%)
I worry vaccines' side effects to be vaccinated	204 (39.5%)	121 (23.4%)	109 (21.1%)	26 (5.0%)	56 (10.8%)
HPV vaccination is only for people who are sexually active	223 (43.1%)	138 (26.7%)	70 (13.6%)	34 (6.6%)	51 (9.9%)
Attitude level of HPV vaccination	Positive	263	51%		
	Negative	253	49%		

**Table 4:** Acceptance of HPV vaccination among girl students in Arba Minch town, Southern Ethiopia, 2020.

Acceptance-related questions of HPV vaccination	Strongly disagree	Disagree	Neutral	Agree	Strongly agree
I do not prefer the existence of HPV vaccination in Ethiopia	208 (40.3%)	99 (19.2%)	51 (9.9%)	67 (13.0)	91 (17.6%)
I prefer to receive HPV vaccination for myself if it is available	52 (10.1%)	54 (10.5%)	76 (14.7%)	146 (28.3%)	188 (36.4%)
I do not advice the HPV vaccination for my sister	114 (22.1%)	19 (23.1%)	147 (28.4%)	69 (13.4%)	67 (13.0%)
I do advice the HPV vaccination for my friends	71 (13.8%)	47 (9.1%)	99 (19.2%)	161 (31.2%)	138 (26.7%)

**Table 5:** The cross-tab result to show the link between religion, ethnic group, and the acceptability of the HPV vaccination among girl students in Arba Minch town, Southern Ethiopia, 2020.

	HPV vaccination		Total
	Not accepting	Accepting	
Religions			
Protestant	172 (55.1%)	140 (44.9%)	312
Orthodox	61 (41.2%)	87 (58.8%)	148
Catholic	7 (35.0%)	13 (65.0%)	20
Muslim	6 (35.3%)	11 (64.7%)	17
Others	10 (52.6%)	9 (47.4%)	19

Ethnic group			
Gamo	191 (51.9%)	177 (48.1%)	368
Gofa	22 (47.8%)	24 (52.2%)	46
Amhara	14 (42.4%)	19 (57.6%)	33
Oromo	13 (56.5%)	10 (43.5%)	23
Wolayta	12 (36.4%)	21 (63.6%)	33
Others	4 (30.8%)	9 (69.2%)	13

**Table 6:** Association between sociodemographic characteristics and other related factors with acceptance of HPV vaccination among primary school girls in Arba Minch town, Southern Ethiopia.

Variables		Acceptance of HPV vaccine		COR (95% CI)	AOR (95% CI)	*P*_ value
		Accepting	Not accepting			
Age in years	13	53 (10.3%)	148 (28.7%)	1	1	1
	14	207 (40.1%)	108 (20.9%)	5.35 (3.62-7.91)	2.93 (1.57_5.47)^∗^	0.001

School type	Public	226 (43.8%)	244 (47.3%)	1	1	1
	Private	34 (6.6%)	12 (2.3%)	3.06 (1.55_6.05)	0.57 (0.21_1.51)	0.259

Childhood immunization	Yes	207 (40.1%)	59 (11.4%)	13.04 (8.58-19.83)	14.85 (8.58_25.72)^∗∗^	≦0.001
	No	53 (10.3%)	197 (38.2%)	1	1	1

Grade level	4 and 5	22 (4.3%)	36 (7.0%)	1	1	1
	6	42 (8.1%)	82 (15.9%)	0.84 (0.44-1.60)	1.01 (0.42_2.45)	0.977
	7	88 (17.1%)	57 (11.0%)	2.53 (1.35-4.73)	0.89 (0.35_2.24)	0.805
	8	108 (20.9%)	81 (15.7%)	2.18 (1.19-3.99)	0.57 (0.22_1.44)	0.231

Fathers educational status	No education	22 (4.3%)	50 (9.7%)	1	1	1
	Primary	30 (5.8%)	59 (11.4%)	1.16 (0.59-2.25)	0.89 (0.35_2.26)	0.802
	Secondary	63 (12.2%)	44 (8.5%)	3.25 (1.73-6.12)	2.03 (0.83_4.97)	0.121
	More than secondary	145 (28.1%)	103 (20.0%)	3.20 (1.83-5.61)	1.39 (0.63_3.05)	0.418

Mothers educational status	No education	24 (4.7%)	88 (17.1%)	1	1	1
	Primary	42 (8.1%)	73 (14.1%)	2.11 (1.17-3.81)	1.86(0.83_4.2)	0.133
	Secondary	64 (12.4%)	41 (7.9%)	5.72 (3.15-10.41)	2.40 (1.01_5.73)^∗^	0.048
	More than secondary	130 (25.2%)	54 (10.5%)	8.83 (5.08-15.33)	3.64 (1.61_8.25)^∗^	0.002

Attitude level	Negative	63 (12.2%)	190 (36.8%)	1	1	1
	Positive	197 (38.2%)	66 (12.8%)	9.00 (6.04_13.41)	5.22 (2.96_9.19)^∗∗^	≦0.001

Knowledge level	Poor	26 (5%)	102 (19.8%)	1	1	1
	Good	234 (45.3%)	154 (29.8%)	5.96 (3.70_9.60)	2.49 (1.19_5.24)^∗^	0.016

Information source	Social media	158 (30.6%)	199 (38.6%)	0.31 (0.17_0.59)	0.66 (0.28_1.54)	0.336
	HCP	64 (12.4%)	42 (8.1%)	0.60 (0.30_1.23)	0.69 (0.27_1.77)	0.436
	Others	15 (2.9%)	38 (7.4%)	1	1	1

Social media: TV, radio, and internet; others: family and friends; HCP: health care providers; COR: crude odd ratio; AOR: adjusted odd ratio; CI: confidence interval, *P* value: probability value. Key notes: ^∗^: statistically significant; ^∗∗^: strongly statistically significant. 1: reference category.

## Data Availability

All data that support this finding were readily available in this manuscript.
